# Supplementary material for: Development of a computer-aided design software for the quantitative evaluation of aesthetic damage
Source: PLoS One. 2019 Dec 18;14(12):e0226322. doi: 10.1371/journal.pone.0226322 (PMC6919621; doi:10.1371/journal.pone.0226322)
Supplement: S1 Code site — 1. (ZIP) [file pone.0226322.s001.zip › [EESC_jr][Projeto]Relatorio_07_21/reports/templates_zip/gentelella-master.zip!/gentelella-master/production/form_buttons.html]

Gentellela Alela! | 


Gentellela Alela!

Welcome,

## John Doe

  


### General

- Home 
  - Dashboard
  - Dashboard2
  - Dashboard3
- Forms 
  - General Form
  - Advanced Components
  - Form Validation
  - Form Wizard
  - Form Upload
  - Form Buttons
- UI Elements 
  - General Elements
  - Media Gallery
  - Typography
  - Icons
  - Glyphicons
  - Widgets
  - Invoice
  - Inbox
  - Calendar
- Tables 
  - Tables
  - Table Dynamic
- Data Presentation 
  - Chart JS
  - Chart JS2
  - Moris JS
  - ECharts
  - Other Charts
- Layouts 
  - Fixed Sidebar
  - Fixed Footer

### Live On

- Additional Pages 
  - E-commerce
  - Projects
  - Project Detail
  - Contacts
  - Profile
- Extras 
  - 403 Error
  - 404 Error
  - 500 Error
  - Plain Page
  - Login Page
  - Pricing Tables
- Multilevel Menu 
  - Level One- Level One
      - Level Two
      - Level Two
      - Level Two
    - Level One
- Landing Page Coming Soon

- John Doe
  - Profile
  - 50%
    Settings
  - Help
  - Log Out
- 6
  - John Smith
    3 mins ago

    Film festivals used to be do-or-die moments for movie makers. They were where...
  - John Smith
    3 mins ago

    Film festivals used to be do-or-die moments for movie makers. They were where...
  - John Smith
    3 mins ago

    Film festivals used to be do-or-die moments for movie makers. They were where...
  - John Smith
    3 mins ago

    Film festivals used to be do-or-die moments for movie makers. They were where...
  - **See All Alerts**


### Form Buttons

Go!

## Default Buttons

- - Settings 1
  - Settings 2

Default
Primary
Success
Info
Warning
Danger
Dark
Link

## Rounded Button

- - Settings 1
  - Settings 2

Default
Primary
Success
Info
Warning
Danger

## Button Dropdown

- - Settings 1
  - Settings 2

Default 

- Action
- Another action
- Something else here
- Separated link

Primary 

- Action
- Another action
- Something else here
- Separated link

Success 

- Action
- Another action
- Something else here
- Separated link

## Split Button Dropdown

- - Settings 1
  - Settings 2

Action


Toggle Dropdown

- Action
- Another action
- Something else here
- Separated link

Action


Toggle Dropdown

- Action
- Another action
- Something else here
- Separated link

Action


Toggle Dropdown

- Action
- Another action
- Something else here
- Separated link

## Button App Design

- - Settings 1
  - Settings 2

Add the class `.btn .btn-app` tag

 Edit

 Play

 Pause

 Save

6
 Notifications

 Repeat

211
 Users

32
 Orders

12
 Inbox

102
 Likes

## Button Sizes

- - Settings 1
  - Settings 2

Large Button
Medium Button
Small Button
  
Large Button
Medium Button
Small Button
  
Large Button
Medium Button
Small Button

## Button Groups

- - Settings 1
  - Settings 2

Left
Middle
Right

Left
Middle
Right

Vertical button groups

Top
Middle
Bottom

Nested button groups

1
2
3

Dropdown  

- Dropdown link 1
- Dropdown link 2
- Dropdown link 3

Multiple button groups

1
2
3
4

5
6
7

8

Group Radio

Option 1

 Option 2

 Option 3


Gentelella - Bootstrap Admin Template by Colorlib
